# Polyvalent human immunoglobulin for infectious diseases: Potential to circumvent antimicrobial resistance

**DOI:** 10.3389/fimmu.2022.987231

**Published:** 2023-01-09

**Authors:** Sigifredo Pedraza-Sánchez, Adrián Cruz-González, Oscar Palmeros-Rojas, José Luis Gálvez-Romero, Joseph A. Bellanti, Martha Torres

**Affiliations:** ^1^ Unidad de Bioquímica, Instituto Nacional de Ciencias Médicas y Nutrición Salvador Zubirán, Mexico City, Mexico; ^2^ Facultad de Ciencias, Universidad Nacional Autónoma de México (UNAM), Mexico City, Mexico; ^3^ Área de matemáticas, preparatoria agrícola, Universidad Autónoma Chapingo, Texcoco, Mexico; ^4^ Departamento de Investigación, Hospital Regional ISSSTE, Puebla, Mexico; ^5^ Immunology Center, Georgetown University, Washington, DC, United States; ^6^ Subdirección de Investigación Biomédica, Instituto Nacional de Enfermedades Respiratorias Ismael Cosío Villegas, Mexico City, Mexico

**Keywords:** antimicrobial resistance, antibiotic stewardship, public health, misuse of antibiotics, human immunoglobulin, infection control, infectious diseases

## Abstract

Antimicrobial resistance (AMR) is a global health problem that causes more than 1.27 million deaths annually; therefore, it is urgent to focus efforts on solving or reducing this problem. The major causes of AMR are the misuse of antibiotics and antimicrobials in agriculture, veterinary medicine, and human medicine, which favors the selection of drug-resistant microbes. One of the strategies proposed to overcome the problem of AMR is to use polyvalent human immunoglobulin or IVIG. The main advantage of this classic form of passive immunization is its capacity to enhance natural immunity mechanisms to eliminate bacteria, viruses, or fungi safely and physiologically. Experimental data suggest that, for some infections, local administration of IVIG may produce better results with a lower dose than intravenous application. This review presents evidence supporting the use of polyvalent human immunoglobulin in AMR, and the potential and challenges associated with its proposed usage.

## Introduction

With the discovery of penicillin by Fleming, antibiotics first appeared as a potential “magic bullet” that could specifically target disease-causing microorganisms without affecting the host; thus constituting a revolution in medicine (1). Soon after, due to the countless lives saved, research advancements led to the discovery of other novel antibiotic classes; thus, the 1950s to 1970s period was considered “the golden era for antibiotics” (2). Many other antimicrobials, for example those directed at fungal and parasitic agents, were also developed during the latter part of the 20^th^ century (3).

Unfortunately, time and experience showed that several microorganisms developed resistance to almost all of the antibiotics discovered during that period, and many of these drugs became obsolete over the past decades (4). The Nobel Prize in Physiology or Medicine in 1945 was awarded jointly to Sir Alexander Fleming, Ernst Boris Chain, and Sir Howard Walter Florey “for the discovery of penicillin and its curative effect in various infectious diseases.” Ironically, during his Nobel Award acceptance lecture, Fleming made the following prophetic statement: *“The time may come when penicillin can be bought by anyone in the shops. Then, there is the danger that the ignorant man may easily underdose himself and, by exposing his microbes to non-lethal quantities of the drug, make them resistant.”*

Antimicrobial resistance (AMR) occurs in nature, independent of human activity, as an evolutionary process caused by environmental pressures introduced by changing conditions. AMR also appears rapidly due to the selective pressure from the overuse of antimicrobial drugs for human medical treatment and animal husbandry to combat or prevent infectious diseases.

Currently, AMR constitutes a global health problem in medicine for both community- and hospital-acquired infections, causing an increase in disease severity and mortality, and leading to a reduction in therapeutic options. The main bacterial species presenting the highest frequency of AMR of medical interest include the following members of the ESKAPE group: *Enterococcus faecium*, *Staphylococcus aureus*, *Klebsiella pneumoniae*, *Acinetobacter baumannii, Pseudomonas aeruginosa*, and *Enterobacteria* spp. (*E. coli, Salmonella* spp., and *Shigella* spp.) (5). Other bacteria with an increased frequency of AMR are gonococci and *Mycobacterium tuberculosis* (see **Table 1**). In 2017, the World Health Organization called upon the global community of nations to direct its attention to the international challenge of AMR, particularly gram-negative bacteria, and to take action against this problem (16). Other microbes of medical importance that cause infections with high resistance to antimicrobials and high mortality associated with AMR include the fungi *Aspergillus* and *Candida* (e.g., *Candida auris)* (17–20).

**Table 1:** Examples of infections with known antimicrobial resistance.

*Microbe*	Common Infections/disease	AMR (antimicrobial resistance) to	References
*Acinetobacter baumannii*	Ventilator-associated pneumonia	Carbapenem	(6)
*Streptococcus pneumoniae*	Otitis media, Sinusitis, Meningitis, Pneumonia	Beta-lactam antibiotics MDR	(7, 8)
*Staphylococcus aureus*	Bloodstream infection	Methicillin-resistant *S aureus* (MRSA)	(9)
*Klebsiella* sp.	Pneumonia, urinary tract infections, bacteremia, liver infections	Carbapenem-resistant. Colistin, Fosfomycin, Aminoglycosides	(10) (11)
*Escherichia coli*	Bloodstream infection Intra-abdominal infections	Cephalosporines, Aminopenicillins, Aminoglycosides ESBL (extended spectrum beta lactamases)	(9) (12)
*Clostridium difficile*	Healthcare-associated diarrhea	MDR strains	(13)
*Mycobacterium tuberculosis*	Lung Tuberculosis	MDR strains Rifampin and Isoniazid resistance	(14)
*Candida auris*	Localized infections (wounds, abdominal cavity, and lungs), candidemia	Voriconazole, fluconazole, MDR	(15)

In addition, microorganisms in the environment and in clinics may develop resistance to multiple classes of antimicrobials, becoming microbes known as “superbugs”. This is not just a laboratory concern, but has become a global health peril. Infections caused by superbugs are associated with high death tolls and increased economic costs, making it imperative to seek alternative therapies to treat those infections (21, 22). This review investigates the potential use of human intravenous polyvalent immunoglobulin (IVIG) as an alternative or adjuvant therapy to avoid AMR in human infectious diseases.

## Alternative strategies for infection control

In a recent study on the global burden of AMR, based on data from 204 countries and territories, there were an estimated 4.95 million deaths worldwide associated with AMR, including 1.27 million deaths directly attributable to bacterial AMR (23). Therefore, there is a need to reduce the effects of AMR while simultaneously permitting the adequate control of infectious diseases in humans, and the performance of productive activities, such as agriculture and livestock, in a balanced way. **Table 2** summarizes the main approaches to avoid or reduce the impact of AMR.

**Table 2:** Strategies to solve the challenge of AMR.

Strategy or approach	Mechanistic principle	Advantages	Disadvantages
**Reducing the use and misuse of available antibiotics**	Avoiding the selection of resistant strains	Simple and cheap strategy. Efficacy of available antimicrobials is prolonged for a longer time	Requires strong education campaigns for general physicians and general public
**Developing new antimicrobials**	Finding drugs that kill microbes or interfere with replication (broad or reduced spectrum, molecular mechanism known)	Target microbes are eliminated, and disease controlled	Time-consuming for its development (several years). Expensive. Quick emergence of resistant strains conducive to the need for new AM drugs
**Modification of preexisting antimicrobials**	Chemical modification of AM compounds, enhancing their antimicrobial activity to bypass AMR	Cheaper and faster development compared to developing new antimicrobials	Emerging resistant strains to the new AM drugs
**Drug repurposing**	Use of preexisting drugs with known effects, with secondary antimicrobial properties	Studies on safety, toxicity, dosing, and pharmacokinetics are done for these compounds	Doses needed for antimicrobial effects may sometimes be very high. AMR can arise in response to repurposed drugs.
**Phage therapy**	Use of bacteriophages (phages) that infect and kill bacteria	Specific phages target and destroy specific bacteria, leaving intact host cells and microbiota. Avoids AMR	Emerging phage resistance
**Antimicrobial peptides**	Usually, the use of small bipolar molecules produced by innate cells (or modified) in animals or other cells in plants with microbe-killing properties	Efficient at eliminating several species of microbes that belong to a family. Avoids AMR	Difficult to target the site of infection and to reach the effective concentration to eliminate microbes
**Vaccination**	Production of immunity to specific microbe-preventable diseases	Proven efficacy to prevent disease and deaths with the massive use of vaccines	Costly and time-consuming to develop
**Use of monoclonal antibodies**	Specific targeting of disease-causing microbes for neutralization or to induce specific killing and immunity	Rapid transfer of immunity. Specificity to the desired molecular microbe target. Avoids AMR	Expensive to develop and to apply treatments
**Use of polyvalent human immunoglobulin**	Poly-specific immunoglobulins obtained from thousands of human donors can potentially recognize, neutralize, and aid in the elimination of microbes causing infection and disease	Rapid transfer of immunity. Enhancement of natural immune responses for specific microbes. Avoids AMR	Expensive, particularly when used at high doses. Difficulty reaching microbes at the site of infection. Insufficient concentration of specific antibodies in the preparation of polyvalent immunoglobulin

Alternative strategies to control antimicrobial drug resistance include: a) a reduction in the use of antimicrobials, b) the development of new AM drugs, and c) the implementation of novel non-antibiotic therapies for infections. Reducing the use of antibiotics is a simple and inexpensive measure for lowering AMR, but it requires a global effort to educate both health care providers and the public. This would dramatically reduce the indiscriminate prescription and demand for antimicrobials (24). A second approach is the development of new antimicrobials, which is time-consuming and costly, from drug discovery to laboratory tests and clinical trials, before a new antimicrobial is licensed for general use in humans (24, 25). The third strategy to find drugs to combat infections, distinct from the existing antimicrobials, includes several options presented below.

Many plant-derived chemical compounds with antimicrobial properties, primarily polyphenols, terpenes, and flavonoids, have shown good results *in vitro* to eliminate diverse bacteria with AMR, as tested alone or in combination with known antibiotics (26). However, controlled clinical trials are warranted. Alternatively, the development of new antimicrobials by chemically modifying existing drugs is relatively faster and less expensive than the development of new AM drugs, but the rapid emergence of strains resistant to the new drugs is very likely. Another strategy to find new antimicrobial drugs is drug repurposing, which allows for the identification of new effects of existing pharmacological drugs for treating humans with a known safety profile but without the investment of cost and time. The discovery of unexpected secondary beneficial effects of some drugs was found by serendipity, but today there are systematic repurposing strategies based on experimental or *in silico* approaches. There are many databases of drugs and their pharmacological effects intended for repository use in cancer, autoimmune diseases, and infectious diseases (27, 28).

Phage therapy is an alternative tool to fight human bacterial infections, distinct from antibiotics, and it consists of the delivery of bacterial-specific lytic viruses (*bacteriophages*, i.e., literally ‘bacterial eaters’, or simply phages). Phages infect bacteria through specific receptors, and then they inject their nucleic acids, followed by the replication of the virus, assembly inside the bacterial cell body and finally the lysis of the bacteria in a host-specific way. The approach is hypothetically ideal because phages are ubiquitous, harmless to all their surroundings and can be administered orally (29). An advantage of phage therapy is that it can leave the beneficial commensal microbiota intact, targeting only harmful bacteria, and phages can be auto renewed while there is a bacterial host available. Phage therapy for infections has been reported in clinical case reports (30, 31) and clinical trials (32), showing variable efficacy against infections (33), and it is currently considered primarily experimental. Additional work is required to establish which are the best infections to treat and the optimal methods to administer potential phage therapies. Another promising non-antibiotic therapy relies on antimicrobial peptides (AMPs), which are antimicrobial agents produced by all living organisms (21). AMPs are amphipathic peptides that can be inserted into the cell wall and cellular membranes of microbes, causing destabilization and disruption (34). Additionally, vaccines constitute a weapon to prevent infectious diseases and antibiotic resistance. The prophylactic use of vaccines allows the host to build an immune response before encountering the pathogen. Thus, following vaccination, infections caused by the occurrence of drug-resistant microbes and superbugs may be less likely to occur (35).

Unfortunately, antibiotic resistance has dramatically increased worldwide in recent decades, with no signs of receding. It adds to the burden on health care and can potentially contribute to the end of the “antibiotic era” (36). Non-antibiotic therapies are promising alternative therapies to combat drug-resistant microbes and superbugs. The discovery of novel classes of antibiotics or alternative therapies requires urgent attention as we move toward a post-antibiotic era (22).

## Mechanisms of action of IVIG and potential use as adjuvant treatment for infections

The use of polyvalent intravenous immunoglobulin (IVIG) constitutes an alternative or complementary therapy to treat infections caused by drug-resistant microbes and superbugs. Commercially available IVIG products are derived from pooled plasma from thousands of healthy donors. These products consist primarily of IgG, and traces of IgA and IgM antibodies (37), which are able to recognize millions of molecules in thousands of microbes (viruses, bacteria, protozoa, fungi, and helminths) due to natural infections or vaccine stimulation in donors (38).

There are two uses of immunoglobulin therapy: replacement therapy, and immunomodulatory therapy. IVIG was produced initially as a replacement therapy for children with primary immunodeficiency (PID) and low levels of IgG (agammaglobulinemia or in severe combined immunodeficiency), and it helped to prevent infections in those children (39). IVIG is usually administered to patients with PID intravenously (IV), between 200 and 600 mg/kg of body weight for restitution therapy every 3-4 weeks. Alternative subcutaneous (SC) administration was performed at a weekly dose of 100 mg/kg of body weight. Most of these therapies are prophylactic. However, the dose may be adjusted depending on the clinical characteristics of the patients: e.g., the frequency and intensity of infections, the level of hypogammaglobulinemia, and the prophylactic use of antibiotics (40). The second use of immunoglobulin is based on its immunomodulatory properties when used in a higher concentration in the treatment of several autoimmune diseases, such as idiopathic thrombocytopenic purpura (ITP), rheumatoid arthritis, systemic lupus erythematosus (SLE), and myelin neurodegenerative autoimmune diseases, such as multiple sclerosis. Higher concentrations of IVIG are required to treat these autoimmune disorders, usually 1 or 2 g/kg every 4 weeks (41–44). However, the great diversity of primary immunodeficiencies, spanning more than 350 diseases caused by mutations in more than 480 genes, is related not only to infection susceptibility but also to autoimmunity, allergy, inflammation, and malignancy (45). Thus, in clinical practice, there is variability in the IVIG dose administered to patients with PID and infections, depending on their pathogenesis and on their clinical manifestations. Although some meta-analysis studies have shown that a higher monthly dose of IVIG is related to a lower risk of infections (46, 47), another meta-analysis study with subcutaneous IgG did not find a significant clinical advantage linked to increasing doses. Therefore, the IVIG dose administered to distinct patients may be individually tailored, with a balance between reducing infections and side effects caused by treatment (48).

There are several mechanisms by which IVIG mediates its antimicrobial effects and modulates the immune response. First, gamma globulin can bind directly to the cell surface membrane or to one of its appendages, e.g., pili or flagella, extirpating the functional activity of the microbe. The mechanisms underlying the beneficial effects of immunoglobulins in infections are thought to involve toxin and superantigen neutralization, bacterial opsonization enhancing phagocytosis, and intracellular killing (49–53). During viral infections, IVIG may neutralize viruses before they infect host cells; in addition, immunoglobulins also recognize viral antigens expressed on the membrane of infected cells and may induce antibody-dependent cell-mediated cytotoxicity by NK cells, which eliminate virus-infected cells (54). Moreover, IVIG also has several immunomodulatory properties. For example, in dermatomyositis, an inflammatory disease, IVIG modulates complement activation mediated by the Fc fraction, binding C3b and C4b fragments, and inhibiting the formation and endothelial deposition of the C5-C9 membrane attack complex (MAC), which causes tissue damage in the microvasculature (55). IVIG also binds and neutralizes the complement anaphylatoxins C3a and C5a *via* the F(ab)´2 fraction, inhibiting the production of histamine and thromboxane by diverse cells; IVIG also protects pigs from the lethal effects of C5a anaphylatoxin (56). In addition, during its manufacture, IVIG is subjected to physicochemical changes, solvents, detergents, and stabilizers to assure sterility and to prevent the formation of IgG aggregates that might activate complement or activate innate immune cells *via* FcγR (57, 58). Other immunomodulator properties of IVIG include the inhibition of autoantibodies by idiotype networks, the saturation of the FcRn receptor (neonatal Fc receptor) aiding in the destruction of autoantibodies, the functional blockade of Fc receptors modulating the activation of leukocytes and cytokine production, and also the influence on the maturation and activation of dendritic cells, macrophages, NK, and other cell populations (59–62). Some of the effector properties of IVIG are modulated by the Fc fraction, while others are modulated by the F(ab)´2 fraction (63). Most of the mechanisms underlying the immunomodulatory effects of IVIG have been studied, primarily, in autoimmune or inflammatory diseases, but they also participate in the inflammation associated with infections. It is beyond the scope of this review to cover all the mechanisms of action of IVIG, so readers interested in a more detailed description of those mechanisms are advised to consult the excellent reviews of the topic (61, 63, 64).

**Figure 1:**
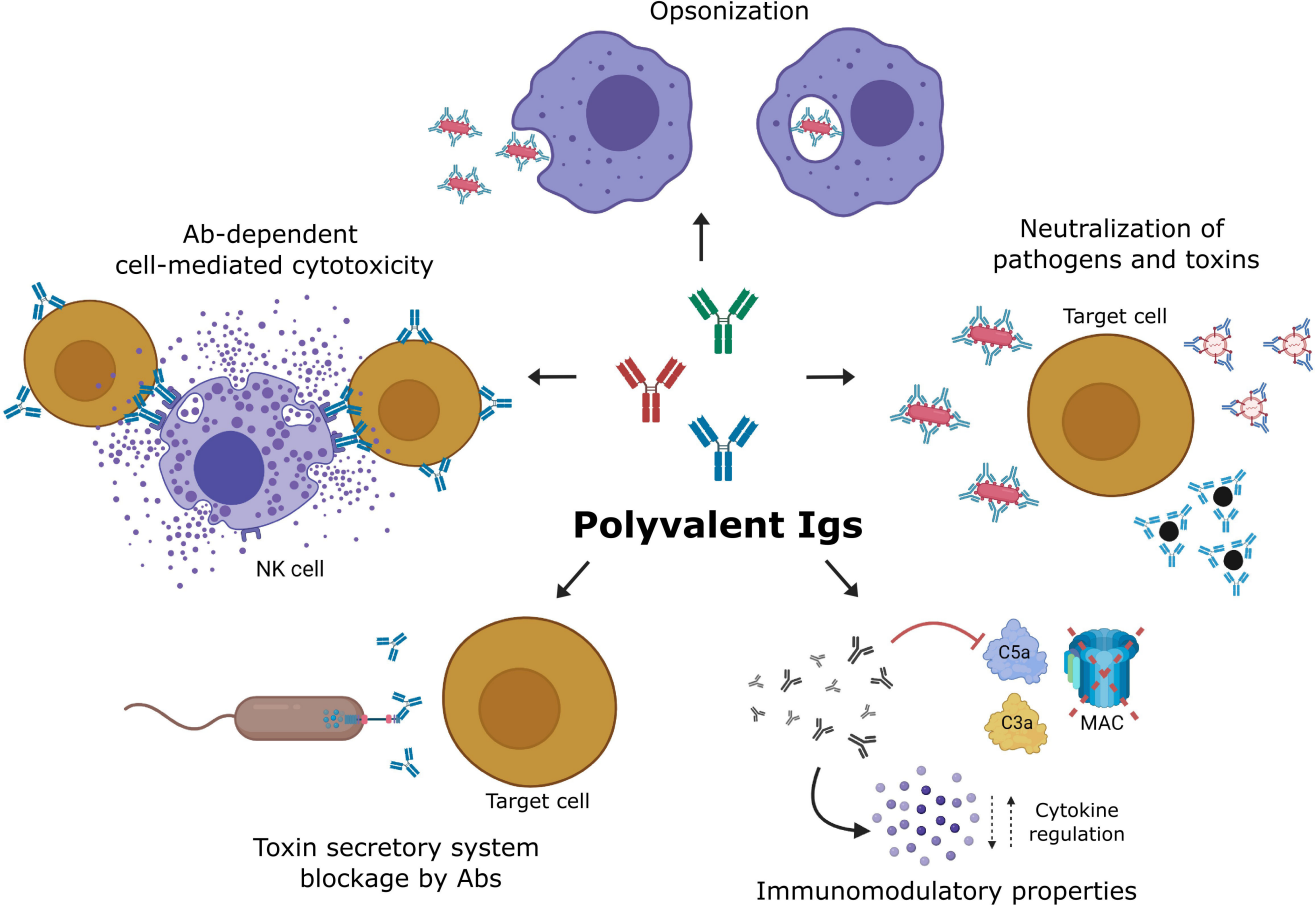
Mechanisms of action of IVIG during the treatment of infections. The neutralization of microbes, toxins, and superantigens blocks the interaction with receptors, preventing their internalization and effects. The opsonization of distinct microbes, such as viruses, bacteria, and fungi, facilitates their phagocytosis and intracellular killing. Antibody-dependent cell-mediated cytotoxicity (ADCC) allows NK cells to kill virus-infected cells that express viral antigens on their membranes. Antibodies of IVIG may also recognize and block components of the Type III toxin secretory system of bacteria, inhibiting toxin injection into host cells. Immunoglobulin molecules in IVIG preparations scavenge activated complement fragments and, thereby, prevent indiscriminate lysis of host cells, immune damage mediated by enhanced opsonophagocytosis, and exaggerated inflammatory reaction. Created with BioRender.com.

Interestingly, many pathogenic gram-negative bacteria, such as *Pseudomonas*, *Yersinia, Salmonella* or *Shigella*, have a special toxin secretory apparatus, Type III, which these bacteria use to actively inject toxins into the cytoplasm of the host cells. This toxin-release mechanism avoids extracellular recognition by antibodies. Antigen V was discovered in *Yersinia pestis* and is a component of the secretion apparatus, which is located on the needle-shaped tip of the bacterial secretion system. IVIG contains antibodies able to recognize and neutralize the antigen PcrV (a homolog of antigen V) from *Pseudomonas aeruginosa*, increasing the survival of *Pseudomonas*-infected mice (65). Therefore, IVIG may have a potential fifth mechanism for bacterial neutralization, which interferes with the type III toxin-secretory apparatus. This fifth IVIG bacterial neutralization mechanism may be functional even for bacteria with AMR (66). **Figure 1** shows the main mechanisms attributed to IVIG for aiding in the control of infections.

The antibodies contained in IVIG promote mechanisms for killing microbes that depend on the enhancement of the normal immune response, even if microbes present AMR. For example, Ono and colleagues (67) experimentally tested the *in vitro* phagocytosis of sensitive or antibiotic-resistant bacteria, including vancomycin-resistant *Staphylococcus aureus* (VRSA), methicillin-resistant *S. aureus* (MRSA), penicillin-resistant *Streptococcus pneumoniae* (PRSP), and extended spectrum beta-lactamase (ESBL) producers *E. coli* and *K. pneumoniae*, by human polymorphonuclear cells (PMN). Opsonization of those bacteria with IVIG increased phagocytosis, and the opsonic indices were quite similar between drug-resistant and drug-sensitive strains. Therefore, the potential of IVIG to control or eliminate microbes that present AMR and superbugs is enormous, making it an attractive treatment option in the post-antibiotic era.

## Laboratory experimental evidence for the use of IVIG against infections, including AMR

The results from *in vitro* studies suggested that IVIG should be considered a viable option to treat diseases caused by bacteria. Human PMNs obtained from patients with septicemia caused by gram-negative bacteria showed an initial reduction in their phagocytic and ROS (reactive oxygen species) production capacity compared to healthy controls. Following *in vitro* opsonization of *E. coli* with IVIG and IgM-enriched IVIG using PMNs from these patients, there was a significant increase in both phagocytosis and ROS production (68). Itoh and colleagues (69) demonstrated that phagocytic activity and killing by human neutrophils against drug-resistant *E. coli* and *P. aeruginosa* was increased in the presence of IVIG and was associated with autophagy. Matsuo and colleagues (70) found that *in vitro* IVIG increases the ROS production and killing of multidrug resistant bacteria by neutrophils isolated from patients receiving immunosuppressive drugs after hematopoietic stem cell transplantation (HSCT).

*In vivo* studies using mouse models have also assessed the efficacy of IVIG against several bacterial infections. For example, Ramisse and colleagues (71) demonstrated that immunosuppressed mice that were challenged intranasally with *S. pneumoniae* and treated with human complete IVIG or with F(ab)´2 were protected against infection. The protective effect was reached at a 50-fold lower concentration of IVIG in mice treated intranasally with whole IVIG or F(ab)´2fragments compared with intravenous administration. In addition, another study showed that intranasal IVIG or F(ab)´2 fragments protected 90% of the mice from lethal nasal influenza (72). In another study, Farag and colleagues (73) infected mice with clinical isolates of MRSA *Staphylococcus aureus via* intraperitoneal injection, and the mice were treated with human IVIG. The mice that received intraperitoneal IVIG had an 80% survival rate, while the rate of survival was only 25% in mice that received intravenous IVIG. Therefore, taken together, these studies suggested that IVIG can be used at lower concentrations, and can be more effective when it is targeted more directly at the infection site, instead of by intravenous administration.

In humans, there is a risk of bacteremia during lung infection and intervention with mechanical ventilation. In murine models of mechanical ventilation and bacterial infection, animals that received human IVIG before *Klebsiella pneumoniae* (74) or *Pseudomonas aeruginosa* (65) challenge had significantly reduced lung infections and bacteremia. These studies suggest the potential use of IVIG to prevent bacteremia in humans during mechanical ventilation.

Other laboratory studies on distinct bacteria have shown the benefits of using only IVIG. Roy and colleagues (75) infected mice intraperitoneally (i.p.) with *Mycobacterium tuberculosis*, and then administered a high i.p. dose of human IVIG to the mice. The animals treated with IVIG had a lower bacterial load in the spleen and lungs than controls that received human serum albumin. Combination therapy using IVIG and antibiotics is another strategy to combat infections caused by drug-resistant bacteria and superbugs. In a murine model, mice were infected intranasally with *Streptococcus pneumoniae*, and were then treated with suboptimal concentrations of ampicillin plus suboptimal IVIG. Mice that received the combined treatment had a significantly higher survival rate and reduction in the bacterial CFU in their lungs and blood compared to those that received individual treatments (76). However, other studies with mice using antibiotics plus human IVIG did not find enhanced bacterial elimination of *Staphylococcus aureus* (77).

In a preclinical study, rabbits were infected with different isolates of *Staphylococcus aureus* that presented AMR and were treated with a 200 mg/kg dose of human IVIG, vancomycin or linezolid, or a combination of IVIG+linezolid or IVIG+vancomycin (78). Rabbits treated with IVIG+linezolid or IVIG+vancomycin had a higher survival rate and lower CFU in their lungs than animals that received individual treatments. The authors of the study demonstrated and concluded that human IVIG contains antibodies that neutralize the *S. aureus* toxins α-hemolysin (HIa) and Panton-Valentine leukocydin (PVL), which are responsible for causing necrotizing pneumonia, and may be used as an adjuvant therapy for human necrotizing pneumonia caused by MRSA.

## Studies in humans using IVIG for microbial infections and infections with AMR

IVIG is used to provide antibodies to patients with PID to prevent infections (79, 80). IVIG has been shown to prevent infections in patients with transient or secondary immunodeficiency, such as transplant patients or cancer patients in radiation treatment, and with infectious diseases that do not respond to conventional therapy, including infections with AMR. **Table 3** presents references for studies using IVIG as an adjuvant treatment in difficult-to-eliminate infections in animal models and humans.

**Table 3:** Some reports on the use of IVIG for the treatment of infections and infections with AMR.

Infection disease or model	Experimental treatment	Control treatment	Variable assessment	Outcome/benefit	Reference
***In vitro* or *in vivo* Experimental models**					
Intranasal *Streptococcus pneumoniae* infection in immunosuppressed mice	Human IVIG or F(ab)´2 by vein or intranasal	PBS	Bacterial CFU in lungs	Significant reduction in lung CFU in mice treated with IVIG. Intranasal TTX w IVIG or F(ab)2´reach effects at 50 times lower concentration vs. vein TTX	(71)
Intranasal *Influenza A virus* infection of Balb/c mice	Human IVIG or F(ab)´2 by vein or intranasal	PBS	Viral load in lungs. Death by influenza pneumonia	Mice treated w/IVIG or F(ab)´2 survived but controls died. Intranasal TTX w IVIG or F(ab)´2protected mice from death at 50 times lower concentration vs. vein TTX	(72)
*In vitro E. coli* infection of human neutrophils from patients with septicemia	*E. coli* + IVIG	*E. coli*	Phagocytosis and ROS production	Phagocytosis and ROS production improvement by IVIG	(68)
*In vitro* infection of human neutrophils with AMR strains of *E. coli* and *P. aeruginosa*	*E. coli* or *P. aeruginosa* + IVIG	*E. coli* or *P. aeruginosa*	Phagocytosis rate and index; Bacterial killing. ROS MPO, Autophagy and NET production	Significant increase in all the assessed parameters, induced by IVIG (including autophagy and bacterial killing, of sensitive or resistant strains)	(69)
Human influenza virus AH1N1 or lethal avian H5N1 virus infection in an animal model with ferrets	Intravenous IVIG or intraperitoneal IVIG prior intranasal viral infection with AH1N1 or H5N1 infection, respectively	Intranasal AH1N1 or H5N1 infection	Viral load in lungs and animal survival	Reduced lung AH1N1 viral load in ferrets that received IVIG. Significant reduced mortality in ferrets that received IVIG and were infected w H5N1 virus	(81)
**Case reports**					
72 y.o. man with *C. difficile* intestinal infection did not respond to vancomycin	Vancomycin + IVIG	Vancomycin for 20 days without improvement	Diarrhea, pain	Diarrhea and pain disappeared at day 6 of IVIG treatment	(82)
Three children with seizures refractory to antiepileptic drugs and encephalitis caused by *Mycoplasma pneumoniae*	IVIG + IV corticosteroids + azithromycin	--	Seizures, hallucinations	Seizures and clinical symptoms of encephalitis disappeared	(83)
55 y.o. man with bacteremia and leg infection with necrotizing group A *Streptococcus* (GAS). STSS	Penicillin, Clindamycin, gentamicin + IVIG (150 mg/kg for 5 days)	--	Fever, edema, erythema in leg and left part of body	After amputation of left leg, hypotension and toxic shock syndrome disappeared. Patient discharged after 25 days in hospital	(84)
57 y.o. woman with diarrhea by *C. difficile* with AMR	IVIG (400 mg/kg) for three days	Metronidazole, vancomycin, clarithromycin, treatments for 6 months	Diarrhea	After IVG treatment, diarrhea ceased, and no relapse was observed over 4 months	(85)
Two 8 y.o. girls from 2 different families with oral candidiasis (P1 with mutation in *IL12RB1* gen. P2 with mutation in *STAT1* gen), and chronic mucocutaneous candidiasis (CMC)	Daily mouthwash three times a day for two weeks	Patient with CMC had severe mouth candidiasis resistant to voriconazole	*C. albicans* CFU in mouth; clinical improvement	*C. albicans* CFU in P1 dropped 95% at day 10 with mouthwash and residual infection was eliminated with nystatin. Infection was reduced by 70% in P2 after 12 days of mouthwash treatment. Infection was eliminated following i.v. caspofungin treatment	(86)
**Preclinical studies and Clinical trials**					
Prevention of infections in 152 adults with multiple traumas, all with mechanical ventilation. RCT Single center.	76 received 36 g IVIG in three doses	76 received a placebo	Pneumonia and sepsis cases at 43 days of following the treatment	Reduction in pneumonia cases in the IVIG group, but sepsis and mortality were similar to control group	(87)
Infection treatment in 56 adult patients with gram-negative septic shock. RCT, Single center	27 received antibiotics + M-IVIG (three days at the beginning of treatment)	28 received antibiotics	Clinical evolution. Death rate at 80 days	Clinical APACHE score significantly improved in M-IVIG group starting at day 5. Mortality: 1/27 in M-IVIG group. 9/28 in the control group	(88)
Infection treatment, 21 adult patients with STSS. Prospective RCT. Multicenter	10 received antibiotics + IVIG (three days)	11 received antibiotics + albumin	Time to shock resolution. Death rate at 28 and 180 days	Study stopped by low recruitment. Mortality at 28 and 180 days: Controls: 36 and 36%. IVIG: 10 and 20%	(89)
Infection treatment, 56 adult Patients with abdominal sepsis. Prospective RCT, Multicenter.	29 received Antibiotics + IgM-enriched IVIG	27 received Antibiotics + Albumin	Clinical evolution, organ dysfunction. Mortality at 30 days	Multiorgan failure was similar in groups. Mortality was 27.5% (IVIG group) and 48.1% in control group	(90)
Preclinical study in a rabbit model. Infection with *S. aureus* with AMR	Infected animals received vancomycin +IVIG or linezolid + IVIG	Infected animals received vancomycin or linezolid	*S. aureus* CFU in lungs. Death rate	*S. aureus* CFU in lungs and mortality were significantly lower in rabbits treated with antibiotics + IVIG	(78)
Infection treatment in 84 adults with invasive group A *Staphylococcus* infections (iGAS). Prospective surveillance of iGAS.	53 receive clindamycin and 14 received additional IVIG	31 received usual treatment, not clindamycin	Death rate at 30 days	Mortality in patients who received clindamycin was 15% compared to 39% in those without clindamycin; mortality was 7% in the IVIG subgroup	(91)
Infection treatment and septic shock in 67 adult patients with STSS	23 received clindamycin or penicillin + IVIG	44 received clindamycin or penicillin	Mortality at 28 days	Mortality was 50% in the control group, while it was 13% in the antibiotics + IVIG group	(92)
**Meta-analysis**					
Infection treatment in adult patients with sepsis	10 clinical trials of studies on sepsis treatment with M-IVIG (pentaglobin)	Control patients received usual care or albumin	Death rate	M-IVIG treatment reduced mortality rate O.R. = 0.35. *Conclusion* M-IGIV TTX benefits patients with gram-negative septic shock	(93)
Adjuvant infection treatment. Critically ill adults with severe sepsis or septic shock	14 clinical trials of studies of adults with sepsis or septic shock, treated with IVIG	Control patients received usual treatment	Death rate	IVIG treatment reduced mortality (O.R. = 0.66), but when only high-quality studies were analyzed, O.R.= 0.96. *Conclusion*: IVIG did not reduce mortality from sepsis or septic shock	(94)
Infection treatment in adult and neonate patients with sepsis or septic shock	Studies with adults: 10 clinical trials on patients who received IVIG; + 7 studies with M-IVIG. Neonate studies: in 5 clinical trials patients received IVIG and in 3 M-IVIG. All patients received usual care + IVIG	Control patients received usual care and treatment, including antibiotics	Death rate	IVIG (R.R. = 0.81) or M-IVIG (R.R.= 0.66) significantly reduced mortality in adults with sepsis, but not in neonates (R.R.= 1.03 for IVIG and R.R. = 0.57 for M-IVIG). Analysis of low-bias risk studies do not show reduced mortality in adults. *Conclusion:* IVIG reduces mortality in adults with sepsis and septic shock, but more RCTs are warranted	(95)
IVIG as prophylactic of CMV and other infections in adult patients with kidney transplantation (KT)	18 clinical studies (8 RCT) prophylactic IVIG for infection (CMV and others)	Control patients received usual care, and in some studies, antivirals	CMV infection, acute graft rejection, graft loss	Prophylactic IVIG marginal reduction on CMV infection (O.R. = 0.68), but not graft rejection (O.R. = 0.96), nor graft loss (O.R. = 1.03). Conclusion: IVIG does not seem to prevent infection in KT	(96)

AMR Antimicrobial resistance. iGAS invasive Group A Staphylococcus. CFU Colony Forming Units. CMC Chronic mucocutaneous candidiasis. CMV Cytomegalovirus. IVIG Polyvalent immunoglobulin G. M-IVIG Polyvalent immunoglobulin enriched on IgM. MPO Myeloperoxidase. NETs Neutrophil extracellular traps. O.R. Odds ratio. RCT: Randomized clinical trial ROS: Reactive oxygen species. R.R. Relative risk. STSS Streptococcal toxin septic syndrome.

*Streptococcus pyogenes* or Group A *Streptococcus* (GAS) is a gram-positive bacterium that causes infections, from benign ones, such as pharyngitis, to severe infections with bacteremia, necrosis and STSS (streptococcal toxin septic syndrome), and that may present with antibiotic resistance (97). In a case report, a 55-year-old man with a leg infection with *Streptococcus pyogenes* did not respond to antibiotics, developed STSS, and his leg was amputated because of severe necrosis. Immediately after surgery, the patient was treated with IVIG for 5 days, with fast improvement and negative *Streptococcus* culture from his blood. He was discharged from the hospital in good condition 25 days after his admission (84).

Patients hospitalized with GAS suffer from severe infections. Clindamycin improves the outcome, but fatal cases are frequent. In a prospective observational study with 84 patients with invasive and severe GAS infections (iGAS), the patients who received clindamycin had a death rate of 15%, while mortality was 39% in patients who did not receive clindamycin. The addition of IVIG to clindamycin treatment reduced the mortality rate to 7%, suggesting an additional benefit of using IVIG (91). In another observational cohort study, of 67 selected patients hospitalized with STSS, 23 received a combination of IVIG + clindamycin and/or penicillin, while 44 received only antibiotics (92). Mortality after 28 days was 7/23 (13%) in the IVIG group, contrasting with 22/44 (50%) in the control group, or conversely, 28-day survival was 87% vs. 50% for the IVIG and control groups, respectively. Accordingly, IVIG increases the probability of surviving STSS. GAS produces a variety of toxins that subvert the innate immune response (97), and IVIG may neutralize those toxins, enabling the normal immune response to eliminate bacteria.

Infections by *Clostridium difficile* produce colitis or diarrhea; they are frequently hospital-acquired and are related to the use of antibiotics. *C. difficile* produces toxins A and B, which cause severe inflammation, namely fluid secretion, and increased mucosal permeability, enteritis and colitis, and these toxins can be neutralized by antibodies contained in IVIG (80). IVIG improved the clinical outcome of child or adult patients with *C. difficile* infection who did not respond to antibiotics, eliminating diarrhea and bacterial toxins in stool (82, 85, 98–100). Therefore, patients suffering GAS or *C. difficile* infections who do not respond to antibiotics can benefit from IVIG treatment, possibly through the neutralization of toxins produced by these bacteria.

The polyvalent nature of IVIG permits the antibody recognition of different microbe families, and it can also be used in fungal or viral infections. *Candida albicans* is an opportunistic fungus that causes infections in patients with primary or secondary immunodeficiency and is sometimes difficult to eliminate. Two girls with primary immunodeficiency affecting IFN-γ and IL-17 production, and with mutations in their *IL12RB1* and *STAT1* genes, were treated for oral candidiasis with IVIG mouthwash. This topical IVIG treatment reduced infection in 12 days at a rate of greater than 95 and 70%, respectively; the patient with the *STAT1* mutation had had chronic mucocutaneous candidiasis since she was 8 months old and severe oral candidiasis resistant to voriconazole. Fungal infections in patients were eliminated after complementary treatments with nystatin and caspofungin (86). Therefore, in that paper, we proposed that *C. albicans* is opsonized in the mouth by IVIG in the mouthwash, which may facilitate the phagocytosis of yeasts by mouth mucosal phagocytes; then, the killing of the fungi occurs by ROS-mediated mechanisms, which circumvents AMR.

IVIG has been tested in clinical trials on hospitalized patients under immunosuppressive interventions to prevent infections or as an adjuvant for infection treatment of sepsis and septic shock. In a comparative study of 150 patients with multiple traumas and mechanical ventilation, 76 patients received IVIG, and 74 received only the usual care. Pneumonia frequency and antibiotic use were lower in the IVIG group than in the control group (28 vs. 43%, p=0.06), but sepsis and mortality were similar in both groups (87). In another study with multiple trauma patients, 21 received IVIG plus penicillin, and 18 received only penicillin. The frequency of pneumonia- and non-catheter related infections was significantly lower in the IVIG group; however, hospital stay and infection-related mortality were similar in both groups (101). IVIG has also been used to prevent cytomegalovirus (CMV) infections in high-risk transplant patients, primarily in kidney transplantation. While some studies reported favorable results both *in vitro* (102) and *in vivo* (103), a meta-analysis study reviewing 18 clinical trials of treatments with IVIG to prevent CMV infections in transplant patients did not find protective evidence (96).

Standard commercial IVIG preparations contain mostly IgG and some traces of IgM and IgA; however, normal human plasma contains IgM and IgA in higher proportions, and IgM is the first isotype of the antibody synthesized during the natural immune response against microorganisms. IgM is a pentameric immunoglobulin that has a high capacity to neutralize toxins, including superantigens, and for agglutination and may also be associated with higher antimicrobial activity (104). The commercial preparation of IgM-enriched IVIG (M-IVIG), which is called pentaglobin, contains 38 g/L IgG, 6 g/L IgM and 6 g/L IgA, while standard IVIG contains more than 96% monomeric IgG, a low percentage of dimeric IgG and traces of IgM and IgA (93). Some studies on sepsis in humans have used M-IVIG instead of standard IVIG. In a one-center study on patients with gram-negative septic shock, 27 were treated with antibiotics plus M-IVIG, and 28 were treated only with antibiotics; the clinical evolution and overall lower mortality (1/27) at 80 days were more favorable for patients in the M-IVIG group than for those in the control group, in which mortality was 9/28 (88). Another multicenter randomized clinical trial (90) on patients with abdominal sepsis also found lower mortality (27.5%) in patients treated with antibiotics plus M-IVIG compared to the control group (48.1%) that received only antibiotics. A meta-analysis by Norrby-Teglund and colleagues on 10 clinical trials of adults with sepsis found favorable results with a mortality reduction in patients treated with the adjuvant use of M-IVIG compared to the usual treatment (O.R. = 0.35), and the results were better in patients with gram-negative sepsis (93). However, a meta-analysis that included 14 clinical trials with patients with severe sepsis or septic shock did not identify a mortality reduction in patients who received IVIG (94).

Alejandria and colleagues (95) published an extensive meta-analysis on studies using polyvalent IVIG to reduce mortality in neonate and adult patients with sepsis and septic shock. The analysis showed that in adults, the use of IVIG (n=1430) or M-IVIG (n=528) reduced mortality compared to non-intervention or a placebo. In contrast, there was no reduction in mortality for neonates with sepsis (n= 3667) treated with IVIG compared to the controls, and better results were observed in studies with M-IVIG, although with fewer patients (3 trials, n=164). The clinical trials on patients with sepsis who received anti-endotoxin antibodies (n=4671) or anti-cytokine monoclonal antibodies did not show significant beneficial effects. The authors highlighted the heterogeneity of the studies (clinical disease, interventions, outcome assessment, and quality of the studies). The sub-analysis of high-quality studies showed a reduction in the beneficial results of IVIG observed in the global analysis. Another disadvantage of using IVIG in sepsis or septic shock is that it must be used during the first stage of infection, and the utility of IVIG is more limited during advanced stages of infection.

In summary, studies on humans with infectious diseases using IVIG as only treatment or as an adjuvant, reported diverse and sometimes contrasting results. Because there is heterogeneity in distinct studies with patients, there is a need to identify the infections and conditions in which the use of IVIG is more promising, as an alternative or as a complementary treatment to avoid AMR.

## Advantages and limitations of IVIG for infectious diseases: The option of immune immunoglobulins and the importance of topical treatments

The first and main advantage of using IVIG to treat infection is that the transfer of immunity is instantaneous. The second is the diversity of antibodies contained in IVIG, with millions of specificities able to neutralize quite diverse toxins, superantigens or to opsonize multiple microorganisms, and provide immunomodulatory properties. The third advantage is to avoid AMR. Thus, the transference of immunity by antibody treatments provides the host with premade effector antimicrobial weapons able to prevent infections or to help eliminate ongoing infections, even if they are caused by microbes with AMR.

Nevertheless, there is an intrinsic lot-to-lot variability in the antibodies present in IVIG that depends, in turn, on the microbes that the plasma donors face during natural infections or vaccination (105); consequently, this variability in the antibody specificities depends on the regions from which donors are selected (106–109). Therefore, a potential constraint for the specific use of IVIG may be that a specific preparation of IVIG does not contain enough titers of antibodies against the target microbe. To overcome this deficiency, the alternative is to obtain or produce immune or specific IgG, which contains high titers of the antibodies of interest. For example, the benefits of immune plasma therapy tested during the beginning of the COVID-19 pandemic were due to high titers of specific antibodies against SARS-CoV-2 coming from subjects who recently recovered from COVID-19 (110, 111). There are also some immune IgGs commercially available for specific needs, such as anti-tetanus, anti-B hepatitis, anti-rabies, anti-RSV or anti-RH (112). Immune immunoglobulins are also obtained through the immunization of human volunteers with specific antigens. Bhakdi and colleagues immunized human volunteers with *S. aureus* α-toxoid and obtained sera from donors to purify and pool the immune IgG. The immune IgG specific for the α-toxin of *S. aureus* showed protection *in vitro* against toxic effects on platelets and monocytes and protected cynomolgus monkeys from the toxic and lethal effects of α-toxin, while normal IVIG did not (113).

During the last century, at the Gamaleya Institute of Moscow, Dr. Simon Skurkovich developed extensive work using human anti-*Staphylococcus* plasma (ASP) and anti-*Staphylococcus* immunoglobulin (ASIG) for treating *Staphylococcus* infections. Dr. Skurkovich immunized human volunteers with *S. aureus* toxoid to obtain ASP and ASIG. The specific immunoglobulin ASIG was used in the USSR and some countries in Western Europe to treat distinct forms of staphylococcal disease, such as meningoencephalitis, pneumonia, osteomyelitis, and post-surgery infections, showing favorable results and a significant reduction in mortality (114). This approach may be clinically useful since staphylococcal infections cause high morbidity and mortality in humans, are frequently present in AMR, and there is no available vaccine for *Staphylococcus* (115). Unfortunately, most of the work by S. Skurkovich and his colleagues was published in Russian and was not known or followed in eastern countries. However, the favorable results obtained in the Soviet Union with ASIG need to be reproduced in clinical controlled studies.

Recently, Emile Jacque and colleagues (116) developed a novel strategy for the enrichment of specific anti-respiratory syncytial virus (RSV) antibodies obtained from human generic IVIG, that is, normal, non-immune sera. Specific antibodies for RSV were purified and enriched by column affinity using protein F from the virus capsid. The enriched anti-RSV Ig efficiently neutralized the virus in an *in vitro* assay with Hep-2 cells. Moreover, anti-RSV Ig given nasally to BALB/c mice inhibited the *in vivo* RSV infection of mice in the lungs and was three times more efficient at protection than Synagis^®^, a monoclonal antibody against RSV (116). Thus, specific anti-RSV Ig antibodies used locally and intranasally protected mice efficiently from infection. Other authors obtained enriched specific anti-*Staphylococcus aureus*, anti-*Streptococcus pyogenes* and anti-*Enterococcus*, using vancomycin-resistant strains, from normal IVIG, and the enriched antibodies were able to neutralize bacteria two to four times more efficiently than normal IVIG and increase phagocytosis by neutrophils (117).

Lastly, giving antibody treatments locally instead of systemically as intravenous injections made the antibodies available *in situ* for the infections, and this approach reduced the concentration of antibodies necessary to give an effector function and to control an infection. The concept of local or topical use of IVIG or specific antibodies has been experimentally tested for infections associated with burns (118–121), peritonitis (122, 123), oral (86), and respiratory infections (71, 72, 76, 124), including SARS-CoV-2 neutralization with an inhaled single-dose of chain fragment variable (small human antibodies) to prevent lung infection (125). In general, the results of the cited papers showed that local delivery of IVIG or specific IgG prevented dissemination of infections to distinct organs and reduced mortality in animal models. The additional benefit of giving IgG at the site of infection or near the infected tissue or organ was the lower concentration of antibodies needed to reach a biological and therapeutic effect. This concept was well illustrated in the experiments by Ramisse and colleagues: the control of *Streptococcus pneumoniae* nasal infection in mice was reached with 50 lower doses of human IVIG administered nasally compared with IVIG given intraperitoneally (71).

## Concluding remarks

The use of IVIG in experimental models and in some human diseases has shown that the antibodies contained in these pools of IgG have the potential to aid in controlling and/or eliminating infections, even when the causal agent exhibited AMR. This potential of IVIG relies on its ability to facilitate, enhance, and modulate mechanisms of the immune response, eliminating microbes and controlling inflammation.

The challenge in optimizing the use of IVIG to fight global AMR consists, firstly, in identifying infections that are more likely to respond to these therapies. Secondly, in using antibodies that are more specific for the infectious agent, and the use of antibodies combined with antibiotics that will increase its efficacy. Finally, finding mechanisms to apply and deliver IVIG at the site of infection has the potential to increase the probability of therapy success and to reduce the amount of IVIG needed to achieve its beneficial effects.

## Author contributions

SP-S: contribution for idea, literature review and draft and edit writing. AC-G: data curation, literature review and draft writing. OP-R, data curation, draft writing and statistical assistance. JG-R, literature review and contribution on clinical aspects; JB, data curation, literature analysis, and manuscript writing and editing; MT, original idea, data curation, resources, supervision, writing and final edits. All authors contributed to the article and approved the submitted version.
